# Alterations of Graphic Properties and Related Cognitive Functioning Changes in Mild Alzheimer’s Disease Revealed by Individual Morphological Brain Network

**DOI:** 10.3389/fnins.2018.00927

**Published:** 2018-12-10

**Authors:** Wan Li, Chunlan Yang, Shuicai Wu, Yingnan Nie, Xin Zhang, Ming Lu, Tongpeng Chu, Feng Shi

**Affiliations:** ^1^College of Life Science and Bioengineering, Beijing University of Technology, Beijing, China; ^2^Department of Biomedical Sciences, Biomedical Imaging Research Institute, Cedars-Sinai Medical Center, Los Angeles, CA, United States

**Keywords:** Alzheimer’s disease, individual morphological brain networks, multiple morphometric features, graph theory, cognitive functioning

## Abstract

Alzheimer’s disease (AD) is one of the most common forms of dementia that has slowly negative impacts on memory and cognition. With the assistance of multimodal brain networks and graph-based analysis approaches, AD-related network disruptions support the hypothesis that AD can be identified as a dysconnectivity syndrome. However, as the recent emerging of individual-based morphological network research of AD, the utilization of multiple morphometric features may provide a broader horizon for locating the lesions. Therefore, the present study applied the newly proposed individual morphological brain network with five commonly used morphometric features (cortical thickness, regional volume, surface area, mean curvature, and fold index) to explore the topological aberrations and their relationship with cognitive functioning alterations in the early stage of AD. A total of 40 right-handed participants were selected from Open Access Series of Imaging Studies Database with 20 AD patients (age ranged from 70 to 79, CDR = 0.5) and 20 age/gender-matched healthy controls. The significantly affected connections (*p* < 0.05 with FDR correction) were observed across multiple regions, both enhanced and attenuated correlations, primarily related to the left entorhinal cortex (ENT). In addition, profoundly changed Mini Mental State Examination (MMSE) score and global efficiency (*p* < 0.05) were noted in the AD patients, as well as the pronounced inter-group distinctions of betweenness centrality, global and local efficiency (*p* < 0.05) in the higher MMSE score zone (28–30), which indicating the potential role of graphic properties in determination of early-stage AD patients. Moreover, the reservations (regions in the occipital and frontal lobes) and alterations (regions in the right temporal lobe and cingulate cortex) of hubs were also detected in the AD patients. Overall, the findings further confirm the selective AD-related disruptions in morphological brain networks and also suggest the feasibility of applying the morphological graphic properties in the discrimination of early-stage AD patients.

## Introduction

Alzheimer’s disease (AD) is one of the most common forms of dementia that has slowly negative impacts on memory and cognition. Eventually, its irreversibility and progression could cause severe neurodegenerative disorders. The late dysconnectivity hypothesis (i.e., the absence of synchronized activity across brain regions) of AD suggests the memory impairments and other cognitive problems are the results of local synaptic disruptions (Arendt, 2009; Gouras et al., 2010). It has been histopathologically defined as an accumulation of β-amyloid (Aβ) plaques (Aβ proteins can exert a toxic effect on surrounding neurons and synapses) and neurofibrillary tangles composed of tau amyloid fibrils (Weiner et al., 2012; Dai et al., 2015).

Recent investigations have exhibited that the cerebral connectomes can be modeled into large-scale brain networks with multiple neuroimaging data and can be further analyzed based on the graph theory (Achard et al., 2006; He et al., 2007; Gong et al., 2009). It allows the quantitative examination of the local and global topological organization of the human brain, such as small world architecture, network efficiency, modularity, and spatial distribution of hubs (Bullmore and Sporns, 2009). So far, AD-related disruptions of brain networks have been reported in both functional and structural research, supporting the hypothesis that AD can be regarded as a dysconnectivity syndrome. For instance, by inspecting the relationship of blood oxygen level dependent (BOLD) signal across brain regions, significantly increased local efficiency but decreased global efficiency have been observed in resting-state functional brain networks of AD patients (Zhao et al., 2012), as well as the reduced modularity (Brier et al., 2014). Meanwhile, diminished clustering coefficient has been found in the AD patients by using cortical thickness covariance networks, which are formed mathematically through the implement of Pearson correlation across regions (He et al., 2008; Li et al., 2012). Reid and Evans (2013) have also reviewed the investigations of morphological networks in AD, and the findings are compatible with functional and pathological reports. Also, AD is one of the brain disorders involved with metabolic distress, which will selectively attack the high-cost components in the entire connectome and lead the network to become a more lattice-like graph (Bassett and Bullmore, 2009; Bullmore and Sporns, 2012). The functional studies have documented that the AD-related disrupted areas yield a highly consensus estimate of default mode network (DMN), which is a set of brain regions that typically deactivate during performance of cognitive tasks (Buckner et al., 2009). Dai et al. (2015) also found the medial and lateral prefrontal and parietal cortices, as well as insula and thalamus are selectively targeted by AD in the entire brain network. The study of diffusion tensor tractography has also detected that the hub regions targeted by AD are predominantly located in the frontal lobe (Lo et al., 2010). Moreover, a thickness-based morphological network study has revealed that AD patients are associated with reduced nodal centrality, revealing the amount of short paths that connected to a node within the network, mostly in the temporal and parietal heteromodal association cortex regions but increases in the occipital cortex regions (He et al., 2008). While, a volume-based morphological study has demonstrated that patients with mild cognitive impairment and AD could retain their hub regions in the frontal lobe but not in the temporal lobe (Yao et al., 2010). Furthermore, the graph-based analysis provides an approach to explore the relationship between network properties and cognitive functioning, which could help researchers to obtain more accurate predictions and diagnoses of AD (Gits, 2016). Such decreased functional connectivity of DMN is found related to the declined cognitive functioning (Binnewijzend et al., 2012), and altered path length of morphological networks in the medial posterior cortex showed the strong relationship with cognitive disruption (Tijms et al., 2013).

Notably, most of these above-mentioned morphological findings are performed in a group-level, and the only AD studies based individual morphological networks are formed with cube-based intensity (Tijms et al., 2013, 2014). Thus, still less is known of AD based on individual morphological brain networks, and multiple morphometric features may provide the further suggestions for lesions identification. The present study used the newly proposed individual morphological brain network to explore the connective anomalies in the early stage of AD. Weighted connections were applied to eliminate the influence of different thresholds. Instead of the conventional way to accomplish the statistical analyses and identify the hubs, the individual effects were considered for the first time rather than by averaging across the intra-group subjects. Cognition was also estimated with graphic properties, including network efficiency, modularity, and betweenness centrality (BC). Here, we hypothesize that network structures of AD patients would be found with alterations concentrated at specific regions. We also posit that graphic properties could explain cognitive functioning to some extent, and provide the further assistance in the future diagnoses of AD in the early stage.

## Materials and Methods

### AD Patients and Control Subjects

The MR imaging data were from Open Access Series of Imaging Studies Database^1^, including 20 early-stage AD patients (10 females and 10 males) aged from 70 to 79 (mean = 73.20 with standard deviation = 2.35) and 20 normal control participants (10 females and 10 males) aged from 70 to 79 (mean = 73.35 with standard deviation = 2.21). The informed and written consents were obtained from each participant (Marcus et al., 2007). The age of subjects in different groups was equally distributed (*p* = 0.84, independent two-sample two-tailed *t*-test) with similar medians (74 in the control group, 73 in the AD group). All the participants underwent ADRC’s full clinical assessment. Dementia status was established and staged using the CDR scale (Morris, 1993; Morris et al., 2001), in which a CDR value of 0, 0.5, 1, 2, and 3 represent no dementia, very mild, mild, moderate, and severe dementia, respectively. Subjects with CDR = 0 and CDR = 0.5 were used for the groups of normal control and AD separately. Cognitive function of all subjects was evaluated using the Mini Mental State Examination (MMSE; Folstein et al., 1975). The AD and control groups had an average MMSE score of 26.3 (range from 20 to 30) and 29 (range from 26 to 30), respectively. For more details on the clinical and demographic information of the participants, please refer to Marcus et al. (2007).

### Image Acquisition

For each participant, three or four individual T1-weighted magnetization-prepared rapid gradient-echo (MP-RAGE) images were acquired using the same 1.5 T Vision scanner (Siemens, Erlangen, Germany) with the following parameters: repetition time = 9.7 ms, echo time = 4.0 ms, inversion time = 20 ms, flip angle = 10°, sagittal orientation with 128 slices, and resolution = 1 mm × 1 mm × 1.25 mm. Multiple T1 images obtained for each subject were motion corrected and then averaged to achieve an image with improved signal-to-noise ratio. For additional details on the post-processing regarding raw images, please refer to Marcus et al. (2007) and Supplementary Materials 1.

### Measurement of Multiple Morphometric Features

**FIGURE 1:**
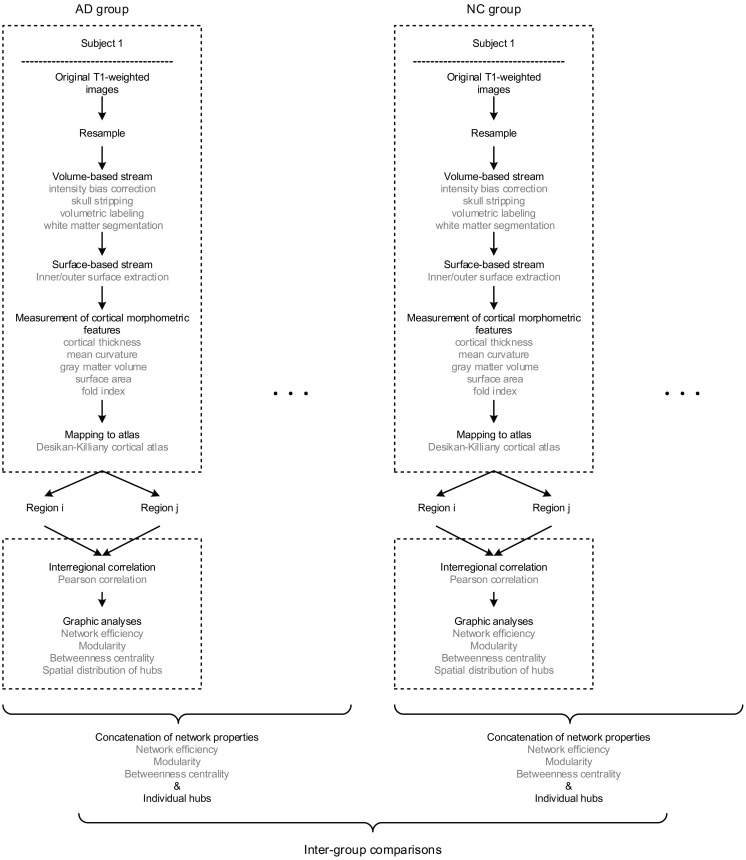
The flow diagram illustrates the pipeline of all the steps involved in the present study. Briefly, for each subject, the raw T1 images were firstly resampled, then processed through volume and surface-based stream in FreeSurfer, and finally obtained regional morphometric feature measurements. Hence, each region was represented as a feature vector. Next, the morphological brain network was built by performing the Pearson correlation across regions. Therefore, every individual has a set of graphic properties, and the inter-group comparisons were conducted by investigating the distinctions of those properties as an intra-group concatenation.

The multiple morphometric features were measured through an image analysis suite, FreeSurfer v5.3.0^2^, whose performance has been endorsed by studies using images acquired from different MRI scanners or sequences (Khan et al., 2008; Shi et al., 2013; Mulder et al., 2014). We applied the program “recon-all” of FreeSurfer to achieve the measurements, whose mathematical background has been described by Dale et al. (1999) and Fischl et al. (1999) in detail. In short, the raw images were firstly resampled into 256 × 256 × 256 with an isotropic resolution of 1 mm × 1 mm × 1 mm. Then, intensity bias correction, skull stripping, volumetric labeling, and white matter segmentation were completed in the volume-based stream. In the following surface-based stream, the inner and outer surface of gray matter were extracted as the gray-white and gray-pial interface separately. Therefore, the cortical morphometric features were measured based on every vertex between the inner and outer surfaces, including cortical thickness (Fischl et al., 1999), mean curvature (Li et al., 2014), gray matter volume, surface area (Panizzon et al., 2009) and fold index (Schaer et al., 2008). Notably, all the computations of surfaces were conducted in the native space, allowing the features as mentioned above to be measured without deformation. Finally, the built-in Desikan-Killiany cortical atlas (Desikan et al., 2006) was applied to obtain the regional measurements, which has the parcellation of 34 regions for each hemisphere based on the structural pattern of the gyrus and sulcus (Supplementary Table 1). The procedure of morphometric features extraction is diagramed in the upper dashed boxes in Figure 1.

### Individual Morphological Brain Network Construction

Above all, it is worth mentioning that there are significant differences existed among the order of magnitudes of morphometric features (10^-1^ to 10^4^). Thus, the z-scores were computed initially (dividing by the standard deviation after subtracting the mean value) for each feature as the standardized values before the network construction. The method of individual morphological brain network based on multiple morphometric features has been described and validated in the previous study (Li et al., 2017) and Supplementary Materials 2. In brief, all the five morphometric features were concatenated into a feature vector for each region. Then, the interconnected matrix was generated by computing the Pearson correlation coefficient for each pair of the feature vectors. Hence, the morphological brain network with 68 nodes and 2278 weighted edges were obtained for each participant. The pipeline of network formation is illustrated in the lower dashed boxes Figure 1.

### Graph Properties Analyses

All the graphic properties were computed using the Brain Connectivity Toolbox (BCT; Rubinov and Sporns, 2010). The calculation of z-score values (see Individual morphological brain network construction) were realized by the normalization function in the statistical analysis software SPSS v22.0 (SPSS Inc., Chicago IL, United States), as well as all the statistical analyses. The visualization was done by the toolkit of BrainNet Viewer (Xia et al., 2013).

#### Network Efficiency

Network efficiency is firstly defined by Latora and Marchiori (2001), which estimates how efficient a network could be to exchange information. On a global scale, efficiency (E_global_) estimates the exchange of information across the entire network where information is concurrently transferred. The local efficiency (E_local_) takes into account the ability of information exchange through the subgraph of the whole connections. We use the program “efficiency_wei” in BCT to realize the computation of efficiency in this study.

#### Network Modularity

A modular structure is revealed by subdividing the network into multiple node communities, with a maximally possible number of intra-module links and a minimally possible number of inter-module links (Girvan and Newman, 2002). The degree to which the network may be subdivided into such delineated and non-overlapping groups is quantified as modularity (*Q*) (Newman and Girvan, 2004). The higher *Q* indicates a strong partition of the network. *Q* was determined as the highest value of rounds (Rubinov and Sporns, 2010). The calculation of *Q* was performed through the program “modularity_und” in BCT.

#### Spatial Distribution of Hubs

Betweenness centrality (BC) is defined as the number of shortest paths between any two nodes that running through the node, indicating the nodal ability of information flow throughout the network (Freeman, 1977). The original BC was normalized to obtain nBC for identifying the hubs by dividing the mean value across the regions. The nodal degree is not used as a descriptor because, in contrast with BC, it only measures the connections linked to the node instead of the shortest path. Hence, the hubs were identified as the nodes with a higher value of nBC, which was more than the summation of the mean and standard deviation for the entire network. Based on the individual brain network, the hubs for each subject were obtained initially. Then the nodes would be recognized as the hubs for the group if more than 30% of subjects in the group have it as a hub. The threshold value of 30% was determined based on the experiments. In this study, the program “betweenness_wei” in BCT was employed to complete the computation of BC.

### Statistical Analyses

Kolmogorov–Smirnov test of the SPSS was used to reveal the normality of each graphic property. Some of the K-S test results indicated a significant departure from a normal distribution for obtained network properties. Fisher’s transformation also failed to realize the conversion to normality. Thus, a rank transformation was applied for network properties values so that the parametric analyses could conduct (Conover and Iman, 1981; Tijms et al., 2013). Ties were replaced by the mean ranks. The significant inter-group variations of E_global_, *Q*, and MMSE scores, as well as E_local_, BC and interregional connections for each region, were revealed by the independent two-sample *t*-tests. Levene’s test for equality of variances was also considered that if the difference of variance falls in the rejection area, the adjusted *p*-value was to be selected. The *t*-test results would be under the false discovery rate (FDR) correction to adjust for the multiple hypothesis testing if necessary (Storey, 2002). The tests were performed at a significance level of 0.05. It is worth to mention that the tests were performed between groups with a concatenation of individual graphic properties. Spearman correlation was also used to assess the relationship between cognitive functioning (MMSE) and graph properties (E_global_, E_local_, mBC and *Q*), which mBC is the average BC across regions for each subject. The influences of age and gender were excluded due to the strong inter-group similarities. The outliers were checked and excluded for all the variables.

## Results

### Aberrant Interregional Connections

**FIGURE 2:**
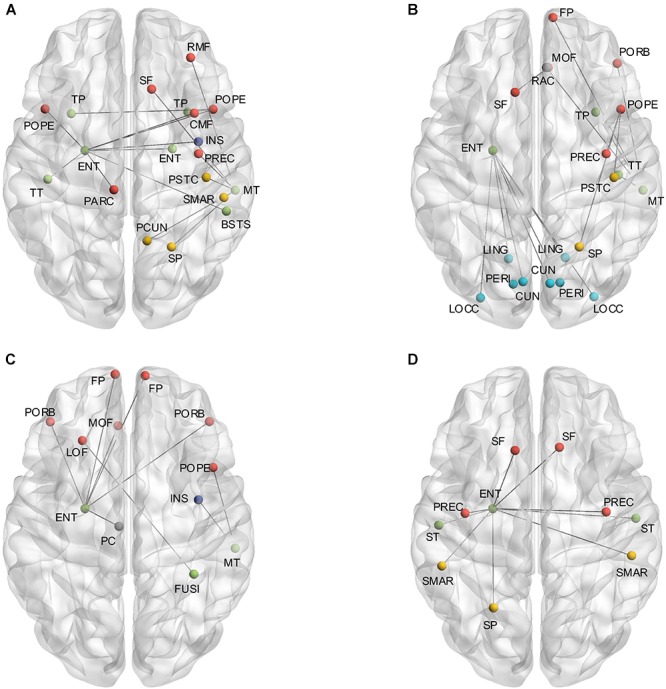
**(A)** Shows the altered interregional connections of decreased positive correlation. **(B)** Illustrates the changed connections of decreased negative correlation. **(C)** Reveals the cases of increased positive correlations, and **(D)** exhibits the decreased negative ones. Colors of the dot are defined to differentiate various lobes: red denotes the frontal lobe, green denotes the temporal lobe, blue denotes the occipital lobe, yellow denotes the parietal lobe, gray denotes the cingulate cortex, and purple denotes the insula.

The independent two-sample one-tailed *t*-tests revealed the significantly altered interregional connections of AD patients with FDR correction (*p* < 0.05). The tests were performed based on the concatenation of individual interregional correlations for each connection (see section “Materials and Methods” and Figure 1). Consequently, 52 out of 2,278 connections were identified with profound variations in the AD patients (illustrated in Figure 2 and listed in Table 1 in the order of decreased positive correlations, decreased negative correlations, increased positive correlations, and increased negative correlations). Broadly speaking, lateralization of anomalies was not detected, and 65.4% of the aberrations showed attenuated correlations in the AD patients (correlation toward to zero). In particular, 17 decreased positive correlations were noticed mostly in the temporal, frontal and parietal lobes (Figure 2A and Table 1). For instance, the connections between the left entorhinal cortex (ENT) and bilateral pars opercularis, and between the middle temporal gyrus and parietal lobe within the right hemisphere. Additionally, 17 attenuated negative correlations were observed in the AD patients (Figure 2B and Table 1), such as the connections between the left ENT and various bilateral regions in the occipital lobe. Moreover, there was 18 connections distinguished with pronouncedly enhanced correlation in the AD patients (9 positives and 9 negatives) (Figures 2C,D and Table 1), predominantly involved with the temporal, frontal and parietal lobes. Furthermore, the widespread altered connections were found to be concentrated in the specific regions, such as the left ENT.

**Table 1:** Alterations of interregional connections between cohorts.

		Correlations		
Regions	Regions	NC	AD	*t*-value
**Decrease in positive correlations**		
ENT_L	INS_R	0.8	0.42	4.49
ENT_L	BSTS_R	0.86	0.5	4.39
ENT_L	CMF_R	0.64	0.18	3.58
TP_L	POPE_R	0.83	0.71	3.22
ENT_L	PARC_L	0.56	0.22	2.75
ENT_L	POPE_R	0.86	0.67	2.54
POPE_R	TP_R	0.79	0.69	2.41
ENT_L	POPE_L	0.79	0.45	2.37
MT_R	PSTC_R	0.3	0.02	2.24
MT_R	SF_R	0.59	0.31	2.24
ENT_L	TT_L	0.71	0.4	2.15
ENT_L	ENT_R	0.97	0.83	2.05
MT_R	PCUN_R	0.33	0.12	1.78
MT_R	SP_R	0.36	0.16	1.43
MT_R	PREC_R	0.37	0.21	1.11
MT_R	RMF_R	0.24	0.12	0.86
MT_R	SMAR_R	0.49	0.4	0.81
**Decrease in negative correlations**		
ENT_L	LOCC_L	-0.97	-0.8	-4.65
CUN_L	ENT_L	-0.59	-0.26	-4.25
ENT_L	LING_L	-0.89	-0.62	-3.71
SF_L	MOF_R	-0.73	-0.38	-3.54
POPE_R	SP_R	-0.73	-0.49	-3.19
ENT_L	CUN_R	-0.55	-0.26	-3.07
ENT_L	LING_R	-0.85	-0.54	-2.93
MT_R	RAC_R	-0.32	-0.04	-2.68
POPE_R	PREC_R	-0.4	-0.1	-2.68
ENT_L	PERI_L	-0.75	-0.45	-2.64
POPE_R	PSTC_R	-0.66	-0.48	-2.54
ENT_L	LOCC_R	-0.93	-0.79	-2.24
ENT_L	PERI_R	-0.75	-0.51	-2.15
MT_R	FP_R	-0.44	-0.25	-1.90
MT_R	PORB_R	-0.42	-0.19	-1.63
MT_R	TP_R	-0.15	0.05	-1.20
MT_R	TT_R	-0.14	-0.06	-0.89
**Increase in positive correlations**		
ENT_L	FP_L	0.25	0.58	-4.02
ENT_L	FP_R	0.3	0.59	-3.50
ENT_L	PORB_L	0.48	0.67	-3.07
ENT_L	PC_L	0.29	0.61	-2.93
ENT_L	PORB_R	0.51	0.72	-2.68
ENT_L	MOF_L	-0.15	0.15	-2.24
MT_R	POPE_R	-0.04	0.19	-2.21
LOF_L	FUSI_R	-0.04	0.05	-0.43
MT_R	INS_R	0.17	0.21	-0.29
**Increase in negative correlations**		
ENT_L	PREC_L	-0.29	-0.55	3.97
ENT_L	SF_L	-0.45	-0.63	3.97
ENT_L	ST_R	0.06	-0.29	3.54
ENT_L	SF_R	-0.49	-0.67	3.19
ENT_L	ST_L	0.06	-0.30	3.07
ENT_L	PREC_R	-0.34	-0.56	2.96
ENT_L	SP_L	-0.75	-0.79	2.15
ENT_L	SMAR_R	-0.42	-0.58	2.09
ENT_L	SMAR_L	-0.42	-0.62	2.05

*The significantly altered connections were listed above in 4 categories (*p* < 0.05, two-sample one-tailed *t*-test with FDR correction). The *t*-test was performed with all the 20 subjects in each group for every connection. The correlations listed above are represented as the average of all the subjects in each group. For each category, the observations are listed in a descendant order of *t*-value. NC denotes normal control*.

### Divergences of Graphic Properties and Cognitive Functioning

**FIGURE 3:**
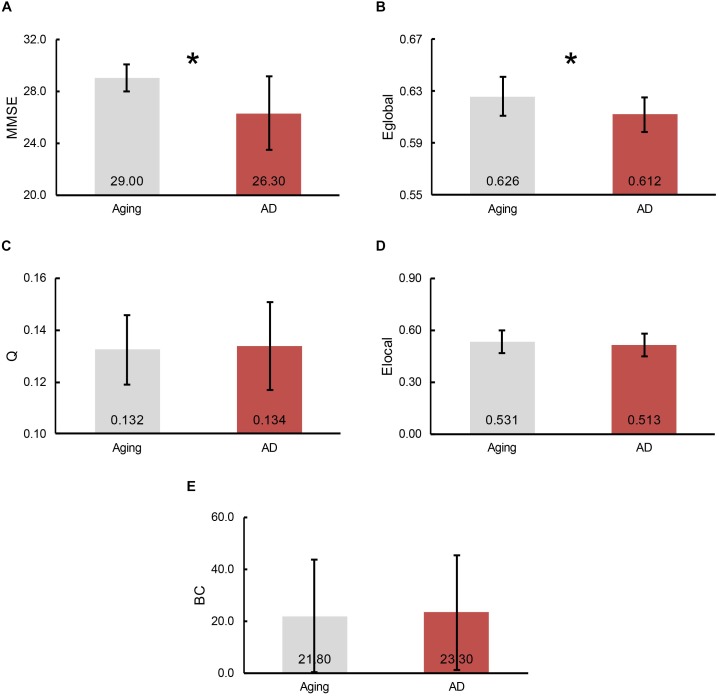
The bars represent the average level of **(A)** MMSE scores, **(B)** global efficiency, **(C)** modularity, **(D)** local efficiency and **(E)** betweenness centrality (BC), separately. The error bar indicates the intra-group deviation. Gray denotes the normal control participants, while red marks the AD patients. The significant differences between cohorts were found in E_global_ and MMSE scores, which indicated by an asterisk.

To demonstrate the divergences of cognitive functioning and multiple graphic properties between normal controls and AD patients, independent two-sample one-tailed *t*-test (*p* < 0.05) was utilized. FDR correction was applied when identifying the remarkable distinction of regional BC and E_local_ between groups. Likewise, the tests were performed based on the concatenation of individual MMSE scores and all the graphic properties for each group. The results of statistical tests revealed a notable decline in MMSE scores in AD patients (*p* = 3.66 × 10^-5^, plotted as the average across subjects in each group, Figure 3A). Additionally, a profound reduction of E_global_ was also detected in the AD patients (*p* = 4.79 × 10^-3^, plotted as the average across subjects in each group, Figure 3B). However, there was no significant inter-group variations observed of *Q* (plotted as the average across subjects in each group, Figure 3C), as well as E_local_ (plotted as the average across subjects and regions in each group, Figure 3D) and BC (plotted as the average across subjects and regions in each group, Figure 3E) for all the regions.

### Cognitive Performance Association

**FIGURE 4:**
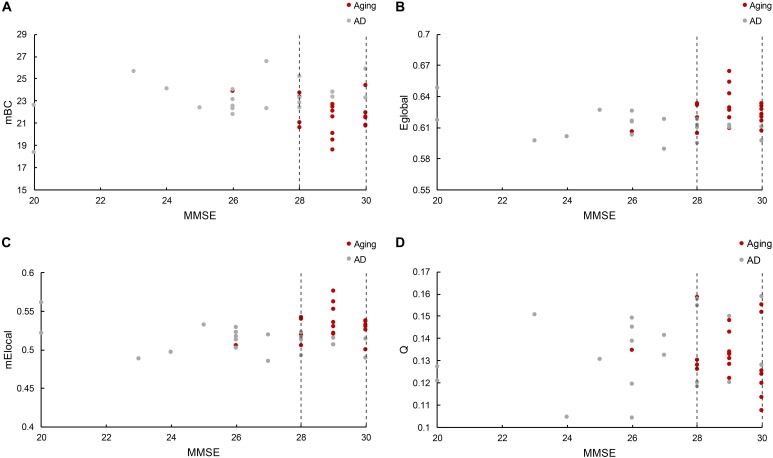
**(A)** Reveals the mBC BC changes according to the MMSE scores, where mBC is the mean BC across regions. **(B)** Shows the global efficiency cases, and **(C)** represents the local efficiency situations based on the MMSE scores, where mE_local_ is the mean E_local_ across regions. **(D)** Illustrates the modularity variations related with MMSE scores. The figure depicts the graphic properties distribution associated with different MMSE scores. Gray dot denotes the normal control participants, while red dot marks the AD patients. The dashed line marked out the higher MMSE score zone between 28 and 30.

The relationship between cognitive functioning and each graphic property was further investigated by using the Spearman correlation coefficient. The results indicated that the MMSE scores were barely related to the modularity (*r* = 0.015), while slightly associated with the E_global_ (*r* = 0.14), mE_local_ (denotes the mean E_local_ across all the regions for each subject, *r* = 0.24) and mBC (stands for the mean BC across all the regions for each subject, *r* = -0.22). But notably, there was only one normal control has the MMSE score lower than 28 (26), and the distribution of graphic properties showed a distinction between groups in the higher MMSE scores. For instance, the mBC of patients were generally greater than the normal controls (Figure 4A), while the E_global_ and mE_local_ of patients were predominantly lower than the controls (Figures 4B,C). The independent two-sample *t*-test further confirmed the significant inter-group differences of mBC (*p* = 0.007), E_global_ (*p* = 0.01), and mE_local_ (*p* = 0.004). Hence, we defined a higher MMSE score zone (from 28 to 30 and marked out with two dashed lines in the Figure 4), and thus MMSE score and graphic properties would be combined as the criteria to identify the AD patients. Specifically, subjects with the lower MMSE score than 28 would be considered as AD patients, and for the subjects with higher MMSE scores, the graphic properties may provide the extra assistance to distinguish the patients from normal controls. Practically, there were 7 out of 8 patients with higher MMSE score distinguished from normal controls based on E_global_, mE_local_, and mBC, separately. The one patient failed to be identified by graphic properties was the same patient with the similar E_global_, mE_local_, and mBC with controls. Additionally, modularity, *Q*, was excluded due to the blurred boundary between cohorts and insignificant statistical analysis results (Figure 4D). The specific MMSE scores and graphic properties of each subject in control and AD group can be found in Supplementary Tables 2, 3, separately.

### Spatial Distribution of Hubs

**FIGURE 5:**
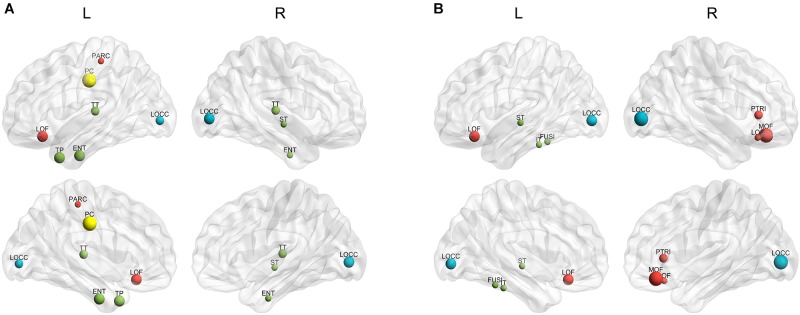
The spatial distributions of hubs for the **(A)** control and **(B)** AD group. Colors of the dot are defined based on the different lobes: red denotes the frontal lobe, green denotes the temporal lobe, blue denotes the occipital lobe, and yellow denotes the cingulate cortex. ENT-entorhinal cortex, FUSI-fusiform gyrus, IT-inferior temporal gyrus, LOCC-lateral occipital gyrus, LOF-lateral orbitofrontal gyrus, MOF-medial orbitofrontal gyrus, PARC-paracentral lobule, PC-posterior cingulate, PTRI-pars triangularis, ST-superior temporal gyrus, TP-temporal pole, TT-transverse temporal gyrus. The size of sphere denotes the value of mean BC across subjects within the group, the higher BC the bigger sphere.

The hubs of each subject were determined as the nodes with greater nBC, and then the most “popular” individual hubs will be selected as the group representations. In the control group, 11 nodes was estimated as the hubs with more than 30% of the subjects have it as a hub individually (Figure 5A and Table 2), including 2 auditory primary regions, 2 paralimbic regions, and 7 unimodal or heteromodal association regions. While in the AD group, 9 nodes were identified as the group hubs with the same criteria mentioned above (Figure 5B and Table 3), including 3 paralimbic regions and 6 unimodal or heteromodal association regions. Those hubs in both groups were predominately located in regions of unimodal or heteromodal association cortex (13 regions), especially in the temporal lobe (9 regions, 6 in NC and 3 in AD), frontal lobe (6 regions, 2 in NC and 4 in AD) and occipital lobe (4 regions, 2 in NC and 2 in AD), Moreover, it was interesting to find that the hubs in the occipital lobe (bilateral lateral occipital gyrus, LOCC) were well retained in the AD patients. But the situations were quite different for the frontal and temporal lobes, that only one hub in the frontal lobe maintained in the AD group (left lateral orbitofrontal gyrus, LOF) and no hub in the temporal lobe persevered. Additionally, the lateralization of hub distribution was changed in the AD patients. For instance, the well-distributed hubs in the temporal lobe transferred into left-lateralization in the AD patients, while the left-sided distribution of hubs in the frontal lobe relocated to the right hemisphere. Furthermore, there were 49 regions identified as hubs with less than 30% of subjects in the control group (5–25%) and 53 regions in the AD group (5–25%). The specific ratios of subjects have these nodes as a hub in control and AD group could be found in Supplementary Tables 4, 5, separately.

**Table 2:** The nodes of the control group with more than 30% of subjects have it as a hub.

Regions	Lobes	Classes	% of subjects	avgBC
Left posterior cingulate	Cingulate cortex	Paralimbic	45%	32.9
Left entorhinal cortex	Temporal lobe	Association	40%	39.1
Left lateral orbitofrontal gyrus	Frontal lobe	Paralimbic	40%	39.1
Left temporal pole	Temporal lobe	Association	40%	28.9
Right lateral occipital gyrus	Occipital lobe	Association	40%	37.4
Left lateral occipital gyrus	Occipital lobe	Association	35%	35.4
Left transverse temporal gyrus	Temporal lobe	Primary (auditory)	35%	31.3
Right transverse temporal gyrus	Temporal lobe	Primary (auditory)	35%	32.5
Left paracentral lobule	Frontal lobe	Association	30%	26
Right entorhinal cortex	Temporal lobe	Association	30%	33.4
Right superior temporal gyrus	Temporal lobe	Association	30%	36.3

*The hub regions of the control group are listed in a descending order of the ratio of subjects. The regions were classified as primary, association, and paralimbic as described by Mesulam (1998). avgBC denotes that BC of each node is averaged across the subjects*.

**Table 3:** The nodes of AD group with more than 30% of subjects have it as a hub.

Regions	Lobes	Classes	% of subjects	avgBC
Right lateral occipital gyrus	Occipital lobe	Association	45%	36
Right medial orbitofrontal gyrus	Frontal lobe	Paralimbic	45%	37.5
Left lateral occipital gyrus	Occipital lobe	Association	40%	44.7
Left lateral orbitofrontal gyrus	Frontal lobe	Paralimbic	40%	39.1
Right pars triangularis	Frontal lobe	Association	35%	38.6
Left fusiform	Temporal lobe	Association	30%	31.1
Left inferior temporal gyrus	Temporal lobe	Association	30%	24.8
Left superior temporal gyrus	Temporal lobe	Association	30%	35.3
Right lateral orbitofrontal gyrus	Frontal lobe	Paralimbic	30%	32.4

*The hub regions of the AD group are listed in a descending order of the ratio of subjects. The regions were classified as primary, association, and paralimbic as described by Mesulam (1998). avgBC denotes that BC of each node is averaged across the subjects*.

## Discussion

By using multiple morphometric features to form the individual morphological brain networks, the present study elucidated the AD-related alterations of cortical connectomes and the relationship between the morphological graphic properties and cognitive functioning for the first time. The analyses of topological structures were conducted based on the concatenation of individual graphic properties, and the definition of group-wise hubs was on account of the number of subjects that harbors the regions as hubs individually. The main findings are as followed: (1) that significantly affected connections were observed across multiple regions, mainly related to the left ENT; (2) that the profoundly changed MMSE score and E_global_ were noted in the AD patients, as well as the pronounced inter-group distinctions of E_global_, mE_local_ and mBC in the higher MMSE score zone; (3) that the reservations and alterations of hubs were both detected in the AD patients. Taken together, the findings further confirm the selective disruption in morphological brain networks of the AD patients, and also indicate the feasibility of applying the morphological graphic properties to provide the auxiliary assistance in the AD diagnosis.

### Interregional Similarities of Morphometric Features

The interregional morphological similarity has been found and repeatedly validated in recent studies based on various morphometric features, such as cortical thickness and regional volume (Lerch et al., 2006; He et al., 2007; Bassett et al., 2008; Sanabria-Diaz et al., 2010). The observation indicates the existence of the interplay of morphometric features across brain regions while disregarding the anatomical distances. Thus, the formation of a connected structure may capture the long-term neurobiological effects (Mechelli et al., 2005). Nevertheless, the underpinning mechanism of this covariance pattern remains elusive. Some conjectures have been debated, such as mutually trophic effects (Ferrer et al., 1995; Aid et al., 2007), environment-related plasticity (Maguire et al., 2000; Draganski et al., 2004; Mechelli et al., 2004), genetic influence (Schmitt et al., 2008), and normal development (Raz et al., 2005; Chen et al., 2011). The axonal tension theory has also been mentioned recently (Tijms et al., 2012; Kong et al., 2015), stating that the interconnected areas are becoming either thicker or thinner as a result of being pulled by a mechanical force (Van Essen, 1997).

### Altered Morphological Graphic Properties

The attenuation of both positive and negative correlations, observed predominantly in the AD patients, may imply the disrupted structural covariation across brain regions (Alexander-Bloch et al., 2013). In particular, the temporofrontal connections, commonly found in the healthy subjects by using intracranial electroencephalographic (Lacruz et al., 2007), were mostly undermined in the AD patients. The magnetoencephalographic study of Hsiao et al. (2014) has also reported the deteriorated synchronization between temporal and frontal lobes in the AD patients, which is implied to be one of the reasons of auditory deviance input. The occipitotemporal connections were observed with significant detriments as well, which is in accordance with the findings of affected white matter connections between occipitotemporal brain regions (Gleichgerrcht et al., 2015). In contrast to the decreases of interregional covariation, enhanced correlations were also noticed in the AD patients. The explanation of these strengthened connections is still poorly understood, but there are some speculations like functional compensation or synchronized atrophy of gray matter in regions under attacked (Alexander-Bloch et al., 2013). Additionally, previous human brain functional studies have examined strong functional correlations between bilaterally homologous regions (Lowe et al., 1998; Hampson et al., 2002; Wang et al., 2006), which may support the phenomenon of simultaneously altered correlations related to the same regions (e.g., left ENT and bilateral occipital and frontal regions, Figure 2).

Moreover, the left ENT was noticed as the most critically influenced region in the AD patients with both decreased and increased correlations, which has also been implicated as a primary site of dysfunction in AD by a convergence of studies (Whitwell et al., 2007; Braak and Del Tredici, 2012; Khan et al., 2014). The function of ENT is generally believed as the essential input of cortical information (the content and context of an experience) to hippocampus (Knierim et al., 2014), and thus may lead the disruption of episodic memory function to be as one of the typical syndromes of AD patients (Nellessen et al., 2015). However, the underlying reason of why the ENT would be attacked the most is not clear yet. A longitudinal study has found that the ENT is not just the “favorite region” of AD, but also shows increased atrophy in the normal aging procedure (Fjell et al., 2012). Therefore, this age-vulnerability presumably renders the regions to be more sensitive to additional, pathological AD-related changes (Fjell et al., 2014). Additionally, selective disruption of structural connectivity in aMCI and AD around ENT has been detected by Mallio et al. (2015), which may allow the conjuncture of increased correlation based on the synchronized alteration related to the ENT. In addition to the ENT, right middle temporal gyrus (MT) was observed as the second most attacked region. The atrophy of MT has also been found in the studies of AD patients with mild syndromes (Convit et al., 2000; Whitwell et al., 2007). Previous functional neuroimaging research has suggested that the MT is involved in several cognitive processes, such as semantic memory (Onitsuka et al., 2004), which has also been detected in the AD patients with loss semantic information (Mårdh et al., 2013).

Furthermore, the determination of group-wise hubs was based on the ratio of subjects who have the region as a hub individually in each group. As listed in Table 2, the maximum owning rate of a hub is only 45% for both groups, which implies the inter-individual difference is an unneglectable factor when analyzing human brain (Kanai and Rees, 2011). It is also in accordance with the findings that one control has a lower MMSE score while one patient has such much similar morphological brain network structure with controls. Additionally, the profound difference of regional BC between groups was not found in the present study (Figure 3E), which may due to the recruiting of subjects, the same age range between cohorts (70–79) and minor aberrations in the early stage of AD. But the inter-group alterations of hubs were noted, such as posterior cingulate cortex (PCC) and ENT, which are compatible with previous studies (Hagmann et al., 2008; van den Heuvel and Sporns, 2013). Evidence has demonstrated the metabolic reduction in the PCC of the AD patients (Minoshima et al., 1997; Liang et al., 2008), and also there is reduced number of fibers derived from PCC to the whole brain (Zhou et al., 2008). Moreover, the atrophy of bilateral ENT may result in the loss of its covariation with other cortical regions (Chan et al., 2001). However, there were also several regions maintained as hubs in the AD group, such as the lateral occipital gyrus (LOCC). Previous studies have indicated that there are regions maintained in the early stage of AD (He et al., 2008), which has also been speculated as disproportionally affected by AD (Engels et al., 2015).

### Association Between Graphic Properties and Cognitive Functioning

The MMSE score has been suggested as an efficient indicator for the AD diagnosis, that it has been broadly applied to determine the cognitive functioning condition of subjects in the multiple research (Hebert et al., 2003; He et al., 2008). In the present study, all the MMSE scores less than 25 were obtained from AD patients, which makes a clear boundary between groups. However, as illustrated in the Figure 4, there are also several patients have higher MMSE scores even compared with the normal controls. Hence, to differentiate the patients with the similar MMSE score from the normal aging especially happened in the early stage of AD becomes a priority. The functional and morphological studies have demonstrated that the cognitive and memory declines in AD patients are associated with the disrupted small-world structure, which is particularly noted as longer characteristic path lengths and larger clustering coefficients compared with the normal aging (Yao et al., 2010; Liu et al., 2012; Brier et al., 2014). The characteristic path length and clustering coefficient reveal the integration and segregation, separately, of the network structure, which also illustrated as the global and local network efficiency (see section “Materials and Methods”). Accordingly, it may back the thought that the morphological network properties of E_global_ and E_local_ could offer extra assistance when MMSE scores are similar between AD patients and controls (Langer et al., 2012). In addition, BC was also found as a sensitive indicator of AD by previous studies (Tijms et al., 2013; Engels et al., 2015). The reason why AD patients could have larger BC in the higher MMSE score zone is unknown, yet we can speculate it as the compensation for the reduced network efficiency so to reach the similar cognitive functioning with the normal controls. Hence, BC may also be able to provide the auxiliary information to improve the accuracy of early-stage AD prediction. Whereas, modularity was failed to distinguish the patients in the present study, even though the functional research shows that AD patients do have the altered brain network structure (Chen et al., 2013). This phenomenon may due to the sensitivity of modularity to the inter-individual difference, which results in the profound varies across subjects both intra- and inter-groups. Overall, the present study indicates that individual morphological brain network properties could be applied as indicators for the early diagnosis of AD, especially with BC and network efficiency.

### Methodological Issues and Future Research

There are several methodological issues noticed in the present study, and they should be addressed and solved in future research.

The interregional connections were measured as the Pearson correlation coefficient in the present study, as enlightened by many earlier studies (He et al., 2007; Chen et al., 2011; Shi et al., 2013). But the observation denotes the summation of the direct and indirect connections across regions, and thus partial correlation has been applied alternatively for eliminating the influence of other regions (Bassett et al., 2008). Notably, the number of variables should be less than the number of samples of each variable in the partial correlation computation. However, the number of regions (i.e., 68 variables) are far beyond the number of morphometric features (i.e., five samples) in the present study. Therefore, the adjusted partial correlation computation may be employed to increase the accuracy of the results of interregional connections in future research.

Moreover, only 20 gender and age-matched subjects were recruited for each group due to the limitation of the database. Hence, how quantitatively the E_global_, E_local_, and BC can differentiate patients from normal controls has also not been thoroughly studied yet. The more convincible results should be based on the broader population with adjustment for age, gender, and education background. Also, different stages of AD have its unique characteristics. Understanding of the entire development progress may offer more information of how the normal aging being disrupted gradually. Accordingly, more AD patients will be recruited with various CDR scores (0.5, 1, 1.5, and 2) as well as the subjects with mild cognitive impairment.

Furthermore, there were only five morphometric features applied in the present study. The reason is that all the five morphometric features have been formerly used to build the morphological brain networks (Li et al., 2017), which implies the feasibility of those features has been verified to represent the morphological connectome structure. Nevertheless, more features could be added to supplement the structural information contained in the connection matrix. Not only the morphometric features but also the features like texture, moments and other general image features could be used in the morphological brain network construction and analysis.

Last, the data used in this research is 1.5T because we want to keep consistency with our previous methodological study (Li et al., 2017). Indeed, it would provide more morphometric information and diagnose assistance when using 3T images to build brain networks. Hence, high-resolution images would be selected priorly in the future study.

## Ethics Statement

The MR imaging data in the present study were selected from Open Access Series of Imaging Studies Database (http://www.oasis-brains.org/). All subjects participated in accordance with guidelines of the Washington University Human Studies Committee. Approval for public sharing of the data was also specifically obtained. The informed and written consents were obtained from each participant.

## Author Contributions

WL and CY proposed the work designing. WL, YN, XZ, ML, and TC processed the data. WL and CY drafted the work. FS helped in revising the draft. SW provided the equipment and suggestions for the work. All authors approved the final version of the manuscript to be published and agreed to be accountable for all aspects of the work in ensuring that questions related to the accuracy or integrity of any part of the work are appropriately investigated and resolved.

## Conflict of Interest Statement

The authors declare that the research was conducted in the absence of any commercial or financial relationships that could be construed as a potential conflict of interest.

## Footnotes

1 http://www.oasis-brains.org/

2 http://surfer.nmr.mgh.harvard.edu/
