# Supplementary material for: Alterations of Graphic Properties and Related Cognitive Functioning Changes in Mild Alzheimer’s Disease Revealed by Individual Morphological Brain Network
Source: Front Neurosci. 2018 Dec 10;12:927. doi: 10.3389/fnins.2018.00927 (PMC6295573; doi:10.3389/fnins.2018.00927)
Supplement: Supplementary file 1 [file Table_1.DOCX]

Table 1. Parcellation of 68 Desikan-Killiany cortical atlas regions and their abbreviations (odd number: left hemisphere, even number: right hemisphere).

| Index | Region | Abbr. | Index | Region | Abbr. |
| --- | --- | --- | --- | --- | --- |
| 1, 2 | Bank of the superior temporal sulcus | BSTS | 35, 36 | Pars orbitalis | PORB |
| 3, 4 | Caudal anterior cingulate | CAC | 37, 38 | Pars triangularis | PTRI |
| 5, 6 | Caudal middle frontal | CMF | 39, 40 | Pericalcarine | PERI |
| 7, 8 | Cuneus | CUN | 41, 42 | Postcentral gyrus | PSTC |
| 9, 10 | Entorhinal | ENT | 43, 44 | Posterior cingulate | PC |
| 11, 12 | Fusiform gyrus | FUSI | 45, 46 | Precentral gyrus | PREC |
| 13, 14 | Inferior parietal | IP | 47, 48 | Precuneus | PCUN |
| 15, 16 | Inferior temporal | IT | 49, 50 | Rostral anterior cingulate | RAC |
| 17, 18 | Isthmus of the cingulate | ISTC | 51, 52 | Rostral middle frontal | RMF |
| 19, 20 | Lateral occipital | LOCC | 53, 54 | Superior frontal | SF |
| 21, 22 | Lateral orbitofrontal | LOF | 55, 56 | Superior parietal | SP |
| 23, 24 | Lingual gyrus | LING | 57, 58 | Superior temporal | ST |
| 25, 26 | Medial orbitofrontal | MOF | 59, 60 | Supramarginal | SMAR |
| 27, 28 | Middle temporal | MT | 61, 62 | Frontal pole | FP |
| 29, 30 | Parahippocampal | PHG | 63, 64 | Temporal pole | TP |
| 31, 32 | Paracentral lobule | PARC | 65, 66 | Transverse temporal | TT |
| 33, 34 | Pars opercularis | POPE | 67, 68 | Insula | INS |
