# Supplementary material for: Alterations of Graphic Properties and Related Cognitive Functioning Changes in Mild Alzheimer’s Disease Revealed by Individual Morphological Brain Network
Source: Front Neurosci. 2018 Dec 10;12:927. doi: 10.3389/fnins.2018.00927 (PMC6295573; doi:10.3389/fnins.2018.00927)
Supplement: Supplementary file 2 [file Table_2.DOCX]

Table 2. The MMSE scores and graphic properties of each subject in the control group.

| Subject No. | MMSE | mBC | Eglobal | Elocal | Q |
| --- | --- | --- | --- | --- | --- |
| 1 | 30 | 20.85 | 0.62 | 0.53 | 0.12 |
| 2 | 29 | 20.09 | 0.64 | 0.55 | 0.13 |
| 3 | 30 | 21.56 | 0.63 | 0.53 | 0.13 |
| 4 | 29 | 21.47 | 0.63 | 0.54 | 0.15 |
| 5 | 30 | 19.50 | 0.65 | 0.56 | 0.12 |
| 6 | 26 | 22.09 | 0.63 | 0.54 | 0.13 |
| 7 | 30 | 23.88 | 0.61 | 0.51 | 0.13 |
| 8 | 29 | 21.94 | 0.62 | 0.53 | 0.12 |
| 9 | 28 | 20.74 | 0.63 | 0.54 | 0.13 |
| 10 | 29 | 20.59 | 0.63 | 0.54 | 0.13 |
| 11 | 29 | 22.68 | 0.62 | 0.52 | 0.13 |
| 12 | 29 | 21.59 | 0.63 | 0.54 | 0.11 |
| 13 | 29 | 22.50 | 0.62 | 0.52 | 0.15 |
| 14 | 28 | 23.74 | 0.60 | 0.51 | 0.13 |
| 15 | 29 | 23.38 | 0.62 | 0.52 | 0.16 |
| 16 | 28 | 21.53 | 0.62 | 0.53 | 0.11 |
| 17 | 30 | 23.79 | 0.61 | 0.51 | 0.13 |
| 18 | 28 | 24.41 | 0.61 | 0.50 | 0.16 |
| 19 | 30 | 18.59 | 0.66 | 0.58 | 0.14 |
| 20 | 30 | 21.09 | 0.63 | 0.54 | 0.13 |
