# Supplementary material for: Alterations of Graphic Properties and Related Cognitive Functioning Changes in Mild Alzheimer’s Disease Revealed by Individual Morphological Brain Network
Source: Front Neurosci. 2018 Dec 10;12:927. doi: 10.3389/fnins.2018.00927 (PMC6295573; doi:10.3389/fnins.2018.00927)
Supplement: Supplementary file 3 [file Table_3.DOCX]

Table 3. The MMSE scores and graphic properties of each subject in the AD group.

| Subject No. | MMSE | mBC | Eglobal | Elocal | Q |
| --- | --- | --- | --- | --- | --- |
| 1 | 28 | 22.85 | 0.61 | 0.52 | 0.12 |
| 2 | 26 | 25.24 | 0.60 | 0.49 | 0.15 |
| 3 | 26 | 23.26 | 0.61 | 0.51 | 0.16 |
| 4 | 27 | 24.03 | 0.60 | 0.50 | 0.15 |
| 5 | 28 | 22.56 | 0.62 | 0.52 | 0.10 |
| 6 | 30 | 22.41 | 0.63 | 0.53 | 0.13 |
| 7 | 20 | 26.56 | 0.59 | 0.48 | 0.13 |
| 8 | 25 | 22.32 | 0.62 | 0.52 | 0.14 |
| 9 | 28 | 22.59 | 0.62 | 0.52 | 0.12 |
| 10 | 27 | 23.29 | 0.61 | 0.51 | 0.13 |
| 11 | 28 | 23.35 | 0.61 | 0.52 | 0.12 |
| 12 | 20 | 25.68 | 0.60 | 0.49 | 0.15 |
| 13 | 26 | 25.88 | 0.60 | 0.49 | 0.16 |
| 14 | 26 | 23.18 | 0.62 | 0.51 | 0.15 |
| 15 | 24 | 21.79 | 0.63 | 0.53 | 0.14 |
| 16 | 30 | 23.82 | 0.61 | 0.51 | 0.15 |
| 17 | 29 | 18.38 | 0.65 | 0.56 | 0.13 |
| 18 | 26 | 22.29 | 0.62 | 0.52 | 0.12 |
| 19 | 29 | 22.41 | 0.62 | 0.52 | 0.12 |
| 20 | 23 | 24.12 | 0.60 | 0.50 | 0.10 |
