# Supplementary material for: Alterations of Graphic Properties and Related Cognitive Functioning Changes in Mild Alzheimer’s Disease Revealed by Individual Morphological Brain Network
Source: Front Neurosci. 2018 Dec 10;12:927. doi: 10.3389/fnins.2018.00927 (PMC6295573; doi:10.3389/fnins.2018.00927)
Supplement: Supplementary file 4 [file Table_4.DOCX]

Table 4. The ratio of subjects that have each node as a hub in the control group. The ratios are between 5% to 25%. avgBC denotes that BC of each node that averaged across the subjects.

| Regions | | % of subjects | | avgBC | | Regions | % of subjects | avgBC |
| --- | --- | --- | --- | --- | --- | --- | --- | --- |
| CMF_L | 25% | | 26.4 | | SF_L | | 10% | 14.8 |
| FUSI_L | 25% | | 33.9 | | SMAR_L | | 10% | 21.9 |
| RMF_L | 25% | | 28.2 | | BSTS_R | | 10% | 20.7 |
| FP_L | 25% | | 23.8 | | CAC_R | | 10% | 15.9 |
| ISTC_R | 25% | | 25.7 | | CMF_R | | 10% | 23.5 |
| PC_R | 25% | | 26.5 | | PORB_R | | 10% | 22 |
| TP_R | 25% | | 24.9 | | PTRI_R | | 10% | 24.9 |
| PHG_L | 20% | | 22.7 | | CUN_L | | 5% | 21.5 |
| ST_L | 20% | | 35.5 | | IT_L | | 5% | 14.6 |
| CUN_R | 20% | | 29.8 | | ISTC_L | | 5% | 22.4 |
| PHG_R | 20% | | 21.8 | | MT_L | | 5% | 11.8 |
| RMF_R | 20% | | 25.1 | | PORB_L | | 5% | 15.9 |
| IP_L | 15% | | 16.4 | | PCUN_L | | 5% | 15.1 |
| LING_L | 15% | | 22.1 | | RAC_L | | 5% | 14.7 |
| MOF_L | 15% | | 25.6 | | SP_L | | 5% | 7.4 |
| PTRI_L | 15% | | 26.2 | | IP_R | | 5% | 17.1 |
| FUSI_R | 15% | | 21.6 | | LING_R | | 5% | 17.9 |
| MOF_R | 15% | | 22.7 | | MT_R | | 5% | 11.6 |
| PARC_R | 15% | | 19.9 | | PERI_R | | 5% | 19.6 |
| POPE_R | 15% | | 22.6 | | PREC_R | | 5% | 15.8 |
| INS_R | 15% | | 21.2 | | RAC_R | | 5% | 16.5 |
| BSTS_L | 10% | | 18.8 | | SF_R | | 5% | 13.2 |
| CAC_L | 10% | | 16.8 | | SMAR_R | | 5% | 19.4 |
| PERI_L | 10% | | 25.7 | | FP_R | | 5% | 16.9 |
| PREC_L | 10% | | 23.6 | |  | |  |  |
