# Supplementary material for: Alterations of Graphic Properties and Related Cognitive Functioning Changes in Mild Alzheimer’s Disease Revealed by Individual Morphological Brain Network
Source: Front Neurosci. 2018 Dec 10;12:927. doi: 10.3389/fnins.2018.00927 (PMC6295573; doi:10.3389/fnins.2018.00927)
Supplement: Supplementary file 5 [file Table_5.DOCX]

Table 5. The ratio of subjects that have each node as a hub in the AD group. The ratios are between 5% to 25%. avgBC denotes that BC of each node that averaged across the subjects.

| Regions | % of subjects | avgBC | Regions | % of subjects | avgBC |
| --- | --- | --- | --- | --- | --- |
| PREC_L | 25% | 28.6 | MOF_L | 10% | 24.5 |
| SMAR_L | 25% | 26.8 | MT_L | 10% | 20.4 |
| TT_L | 25% | 27.7 | PERI_L | 10% | 18.2 |
| BSTS_R | 25% | 21.6 | FP_L | 10% | 22 |
| CAC_R | 25% | 35.3 | INS_L | 10% | 18.7 |
| CUN_R | 25% | 23.9 | IP_R | 10% | 17.3 |
| PHG_R | 25% | 26.5 | LING_R | 10% | 17.5 |
| PC_R | 25% | 30 | PARC_R | 10% | 19.9 |
| ST_R | 25% | 34.7 | RMF_R | 10% | 30.9 |
| CMF_L | 20% | 37.1 | TP_R | 10% | 20.7 |
| LING_L | 20% | 27.2 | BSTS_L | 5% | 18.3 |
| POPE_L | 20% | 25.5 | CUN_L | 5% | 15.9 |
| TP_L | 20% | 28.1 | PORB_L | 5% | 16.6 |
| ENT_R | 20% | 26.9 | PSTC_L | 5% | 13.5 |
| POPE_R | 20% | 26.1 | PC_L | 5% | 19.6 |
| ENT_L | 15% | 26.2 | RMF_L | 5% | 19 |
| ISTC_L | 15% | 27.4 | SF_L | 5% | 17.4 |
| PARC_L | 15% | 23.8 | CMF_R | 5% | 21.2 |
| PTRI_L | 15% | 14.7 | MT_R | 5% | 15.1 |
| RAC_L | 15% | 21.2 | PORB_R | 5% | 15.9 |
| SP_L | 15% | 15.2 | PREC_R | 5% | 21.4 |
| FUSI_R | 15% | 24.7 | PCUN_R | 5% | 13.7 |
| IT_R | 15% | 17.2 | SMAR_R | 5% | 13.4 |
| ISTC_R | 15% | 22.2 | FP_R | 5% | 18.6 |
| PERI_R | 15% | 31.9 | TT_R | 5% | 21.2 |
| SF_R | 15% | 24.9 | INS_R | 5% | 17.2 |
| CAC_L | 10% | 16.4 |  |  |  |
