# Supplementary material for: Alterations of Graphic Properties and Related Cognitive Functioning Changes in Mild Alzheimer’s Disease Revealed by Individual Morphological Brain Network
Source: Front Neurosci. 2018 Dec 10;12:927. doi: 10.3389/fnins.2018.00927 (PMC6295573; doi:10.3389/fnins.2018.00927)
Supplement: Supplementary file 6 [file Data_Sheet_1.docx]

Supplementary Materials

1. For each subject, the individual scan files were converted from Siemens proprietary IMA format into 16-bit Analyze 7.5 format using a custom conversion program. Sensitive header fields (patient ID, experiment date) were left blank. Identifying facial features were then removed from the images. This method first masks out all nonbrain voxels in the image, iteratively dilates and smoothes the mask, and finally applies the enlarged mask to the original image to safely remove voxels outside the cranial vault.


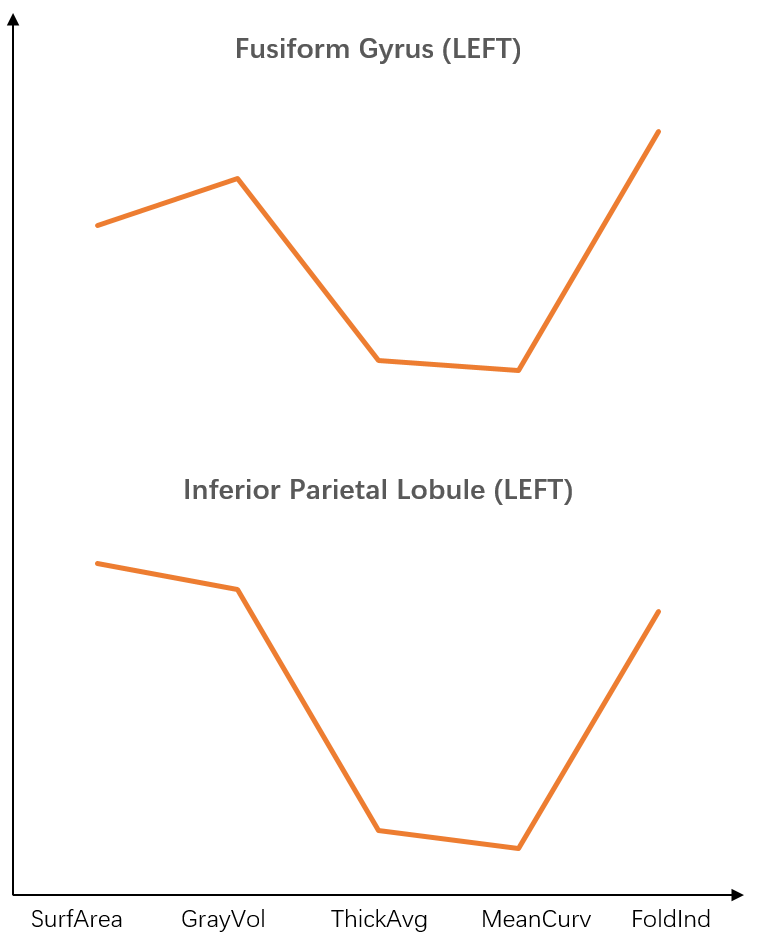


1. The figure above shows the essential idea of individual morphological brain network applied in the present study. For a randomly selected subject, the left fusiform gyrus and left inferior parietal lobule exhibits strong correlation (r=0.91). Functionally, the inferior parietal lobule has been found involved in the perception of emotions in facial stimuli^[[1]](#footnote-1)^. While the fusiform face area, a part of the fusiform gyrus, plays a role in the human visual system that is specialized for facial recognition^[[2]](#footnote-2)^. Hence, the synchronized alterations of morphometric features across brain regions are consistent with the functional and behavioral findings, which indicates the utility and feasibility to use multiple morphometric features to build a brain network.

1. Radua J, Phillips M L, Russell T, et al. Neural response to specific components of fearful faces in healthy and schizophrenic adults[J]. Neuroimage, 2010, 49(1): 939-946. [↑](#footnote-ref-1)
2. Ghuman A S, Brunet N M, Li Y, et al. Dynamic encoding of face information in the human fusiform gyrus[J]. Nature communications, 2014, 5: 5672. [↑](#footnote-ref-2)
